# Rational Design of an Orthogonal Pair of Bimolecular RNase P Ribozymes through Heterologous Assembly of Their Modular Domains

**DOI:** 10.3390/biology8030065

**Published:** 2019-08-31

**Authors:** Yuri Nozawa, Megumi Hagihara, Md Sohanur Rahman, Shigeyoshi Matsumura, Yoshiya Ikawa

**Affiliations:** 1 Department of Chemistry, Graduate School of Science and Engineering, University of Toyama, Gofuku 3190, Toyama 930-8555, Japan; 2 Graduate School of Innovative Life Science, University of Toyama, Gofuku 3190, Toyama 930-8555, Japan

**Keywords:** modular engineering, ribonuclease P, ribozyme, tRNA processing

## Abstract

The modular structural domains of multidomain RNA enzymes can often be dissected into separate domain RNAs and their noncovalent assembly can often reconstitute active enzymes. These properties are important to understand their basic characteristics and are useful for their application to RNA-based nanostructures. Bimolecular forms of bacterial RNase P ribozymes consisting of S-domain and C-domain RNAs are attractive as platforms for catalytic RNA nanostructures, but their S-domain/C-domain assembly was not optimized for this purpose. Through analysis and engineering of bimolecular forms of the two bacterial RNase P ribozymes, we constructed a chimeric ribozyme with improved catalytic ability and S-domain/C-domain assembly and developed a pair of bimolecular RNase P ribozymes the assembly of which was considerably orthogonal to each other.

## 1. Introduction

In the molecular evolution of protein enzymes, the emergence of more complex structures through gene fusion and less complex structures through gene fission are important events in the expansion of the diversity of protein structures and functions [1,2]. Gene fusion usually produces a multidomain protein with a single polypeptide chain. The resulting protein exhibits biological functions carried by two (or more) fused polypeptide chains. In contrast, gene fission generates a pair (or a set) of smaller proteins, each of which originally acted as a structural domain in a parent multidomain protein. Complex functions of the parent multidomain proteins can often be reconstituted through the noncovalent assembly of the smaller proteins generated by gene fission [1]. Such reconstitution has been applied to design protein-based biosensors and nanomaterials [3,4,5,6,7], which include self-assembling protein hydrogels and supramolecular protein polymers the design of which depends on artificial gene (re)fusion of the domain proteins [7].

**Figure 1:**
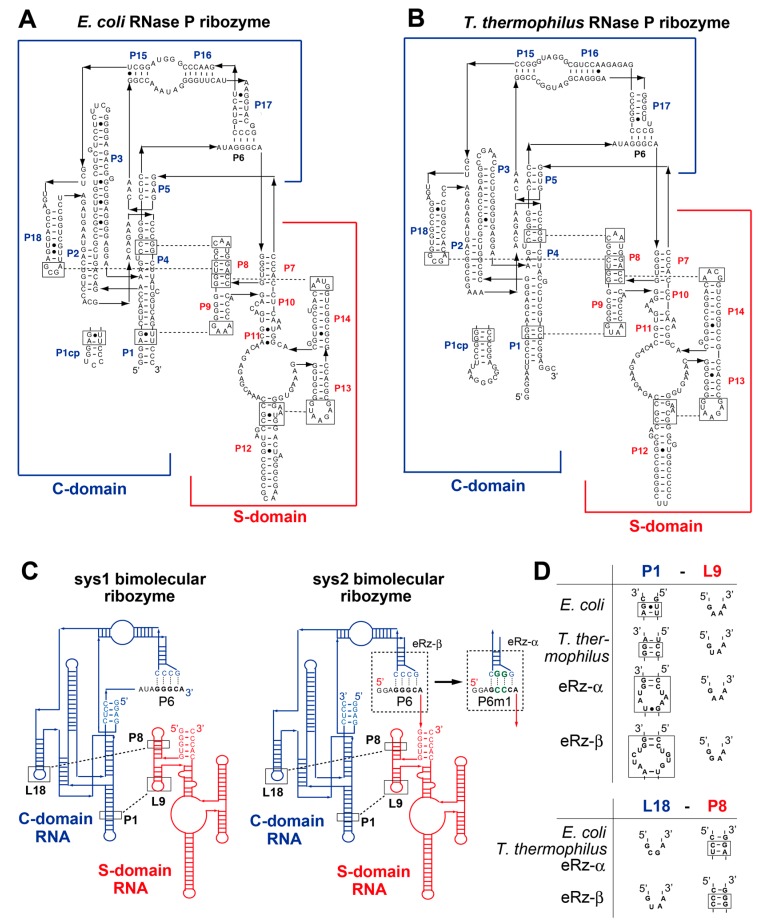
Secondary structures of *Escherichia coli* and *Thermus thermophilus* A-type RNase P ribozymes. (**A**, **B**) Nucleotide sequences and secondary structures of the *E. coli* (**A**) and *T. thermophilus* (**B**) RNase P ribozymes. Arrowheads superimposed on solid lines indicate 5′-to-3′ polarity. Boxes with solid lines indicate elements participating in tertiary interactions, which are shown as broken lines. P1 cp indicates a loop element introduced for circular permutation in each ribozyme. (**C**) Schematic secondary structures of the sys1 (left) and sys2 (right) bimolecular ribozymes. Sys1 bimolecular ribozymes were derived from the circular permutants of the parent ribozymes by dissecting them at the J6/7 and J7/5 junctions. Sys2 ribozymes commonly have base substitutions at J5/6 elements, which connect P5 and P6 and serve as the 5′-end of the sys2 S-domain RNA. J5/6 elements, which are 5′-AUA-3′ in the parent ribozymes, were replaced with 5′-GGA-3′ to improve the efficacy of in vitro transcription. This substitution also did not alter the catalytic abilities of the sys2 ribozymes. Broken lines indicate RNA–RNA tertiary interactions supporting assembly of the S-domain and C-domain. In the sys2 format, P6 base pairs serve as interdomain interactions, which can provide orthogonal P6m1 base pairs. (**D**) Tetraloop–receptor tertiary interactions that support assembly between the S-domain and the C-domain.

In natural evolution as well as artificial engineering of functional RNAs with three-dimensional (3D) structures, fusion or fission of the RNA strands must generate multidomain RNAs or smaller structural RNAs, respectively. Smaller structural RNAs can also reconstitute the parent multidomain structures and their functions through noncovalent assembly [8,9,10,11,12,13,14,15]. Bacterial RNase P RNAs, the structures and functions of which have been studied extensively, constitute a typical class of multidomain ribozymes [16,17,18,19]. The catalytic ability of bacterial RNase P RNA plays a central role in the 5′ maturation of precursor tRNAs (pre-tRNAs). Bacterial RNase P ribozymes commonly have a modular architecture consisting of two large structural domains (Figure 1). Both domains preserve their secondary and tertiary structures after physical dissection of the two domains into two structured RNAs [13,14]. The larger domain is called the catalytic domain (C-domain) because it contains structural components that participate directly in the promotion of hydrolytic cleavage of a phosphodiester bond in substrate pre-tRNAs [16,17,18,19]. The smaller domain is called the specificity domain (S-domain) because it confers substrate specificity on pre-tRNAs. Active RNase P ribozymes can be reconstituted by noncovalent assembly of C-domain RNA and S-domain RNA [13,14].

The resulting bimolecular RNase P ribozyme has also been proposed as a platform to generate supramolecular ribozyme polymers (Figure S1) [20]. Such RNA-based supramolecular assembly can be a promising class of RNA-based functional nanomaterials and may also have existed as an ancient form of membraneless organelle in the RNA world [21,22]. Functional reconstitution of RNase P ribozymes has been reported for the *Escherichia coli* and *Bacillus subtilis* ribozymes [13,14]. These have been used as model ribozymes for biochemical studies because they represent two major subclasses of bacterial RNase P ribozymes, i.e., A-type and B-type, to which the *E. coli* ribozyme and *B. subtilis* ribozyme belong, respectively [16,17,18]. They were also the first candidates as structural platforms to design supramolecular RNase P RNA-based nanostructures with catalytic functions (Figure S1) [20]. Preliminary analysis, however, revealed that the assembly between C-domain and S-domain in the *E. coli* bimolecular ribozyme is not strong enough to construct supramolecular ribozyme polymers (Figure 1A) [20]. Protein-based and RNA-based enzymes in thermophilic bacteria have been used for structural studies [23,24,25,26]. In this study, we first examined an RNase P ribozyme from thermophilic bacterium *Thermus thermophilus* as an alternative candidate to the *E. coli* ribozyme [27]. However, we found that a chimeric bimolecular ribozyme consisting of the *E. coli* C-domain RNA and the *T. thermophilus* S-domain RNA was more promising. We also developed a rational engineering approach to further improve the assembly and catalytic ability of the resulting chimeric ribozyme.

## 2. Materials and Methods

### 2.1. Plasmid Construction and RNA Preparation

DNA fragments encoding sequences of the full-length *E. coli* RNase P RNA and the full-length *T. thermophilus* RNase P RNA were obtained by PCR amplification with genomic DNAs from *E. coli* JM109 and *T. thermophilus* HB8 as templates, respectively. Amplified DNA fragments were then inserted into *Eco*RI–*Hin*dIII sites of the pUC18 vector. These plasmids were then used to construct circular permutants of the two ribozymes by dissecting them at the junction between P5 and P6 elements and capping the P1 elements with short terminal loops. The sequences of the constructed plasmids were confirmed using a DNA analyzer (4300; Li-COR, Lincoln, NE, USA). The resulting circular permutants then served as templates for PCR to prepare DNA fragments encoding S-domains and C-domains and as template DNAs for in vitro transcription. The T7 promoter sequence was attached by PCR with a T7 promoter-containing sense primer. Transcription reactions were performed for 4.5 h at 37 °C in the presence of nucleotide triphosphates (1 mM each) and Mg^2+^ ions (15 mM). The DNA template in the reaction mixture was removed by DNase treatment for 30 min. The transcribed RNA was purified on 4% denaturing polyacrylamide gels. The published protocol was used for 3′-end labeling of RNAs with BODIPY fluorophore [28].

### 2.2. Ribozyme Activity Assay

An aqueous solution containing the S-domain RNA and the C-domain RNA (final concentrations: 1.0 μM each or 25 nM each) was heated at 85 °C for 5 min. An aqueous solution of the human tyrosyl pre-tRNA (final concentration: 0.5 μM or 250 nM) [29], the 3′-end of which was labeled with BODIPY fluorophore, was also heated at 85 °C for 5 min. The 3× concentrated reaction buffer (final concentrations: 50 mM Tris-HCl, pH 7.5, 1 M KCl, and 50 mM MgCl_2_) was added to each RNA solution and incubated at 37 °C for 30 min. The site-specific cleavage reaction of pre-tRNA was initiated by mixing the two RNA solutions and allowed to proceed at 37 °C. Aliquots were taken at given time points and treated with the same volumes of stop solution containing 75% formamide, 0.1% xylene cyanol, and 100 mM EDTA. The mixtures were electrophoresed on 9% denaturing polyacrylamide gels. After electrophoresis, gels were analyzed with a Pharos FX fluoroimager (Bio-Rad, Hercules, CA, USA). All assays were performed at least twice. The mean values are indicated in the figures with the minimal and maximal values shown by error bars.

### 2.3. Gel Electrophoresis Mobility Shift Assay (EMSA)

An aqueous solution containing the S-domain RNA and the C-domain RNA (final concentrations: 1.0 μM each) was heated at 85 °C for 5 min. To this solution was added 10× concentrated folding buffer (final concentrations: 80 mM Tris-acetate, pH 7.5, and 50 mM Mg(OAc)_2_). The resulting mixture was incubated at 37 °C for 30 min and then at 4 °C for an additional 30 min. After addition of 6× loading buffer containing 50% glycerol and 0.1% xylene cyanol, the samples were loaded onto a 5% nondenaturing polyacrylamide gel (39:1 acrylamide:bisacrylamide) containing 70 mM Tris-borate (pH 8.3) and 50 mM Mg(OAc)_2_. Electrophoresis was carried out at 4 °C, 200 V for the initial 5 min, followed by 75 V for 5 h. A Pharos FX fluoroimager (Bio-Rad) was used to visualize and analyze the resulting gels. Preliminary assay showed that the BODIPY moiety did not interfere with the catalytic activities of bimolecular RNase P ribozymes. The BODIPY moiety, however, slightly affected the mobility of some bimolecular complexes depending on the RNA component labeled by the fluorophore (see lanes 12 and 13 in Figure 4D and also lanes 9 and 10 in Figure 6C).

## 3. Results

### 3.1. Optimization of the Bimolecular RNase P Ribozymes by Domain Chimera

**Figure 2:**
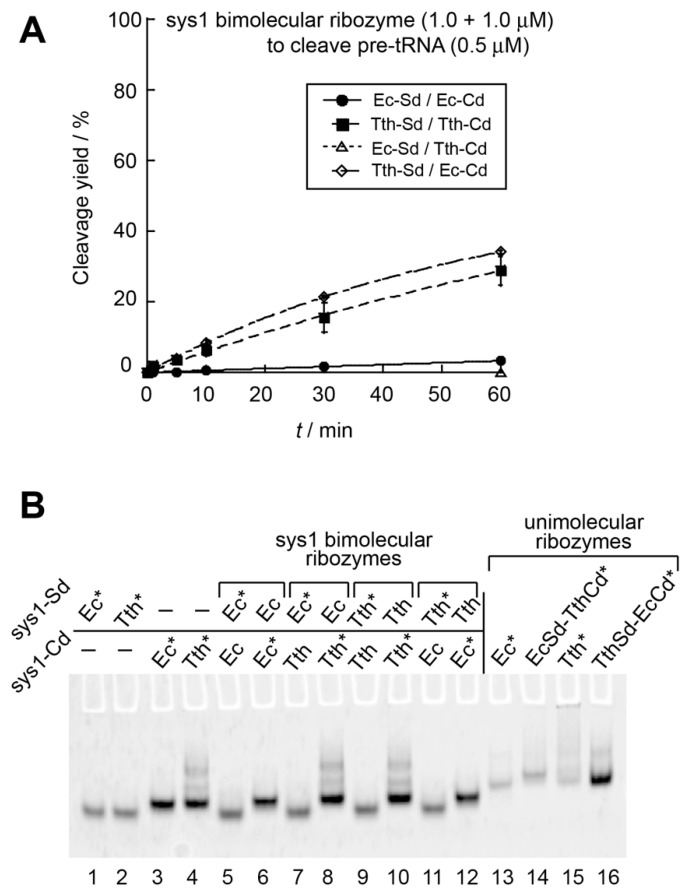
Catalytic ability and interdomain assembly of the sys1 bimolecular ribozymes. (**A**) Site-specific cleavage reactions of pre-tRNA catalyzed by the sys1 bimolecular RNase P ribozymes. Reactions were performed in the presence of 0.5 μM pre-tRNA, 1.0 μM S-domain RNA, and 1.0 μM C-domain RNA. (**B**) Electrophoretic mobility shift assay (EMSA) of S-domain/C-domain complexes in the presence of 50 mM Mg^2+^. Unimolecular RNase P ribozymes were used as size markers for the complexes. Asterisks indicate RNAs labeled with the BODIPY fluorophore.

**Figure 3:**
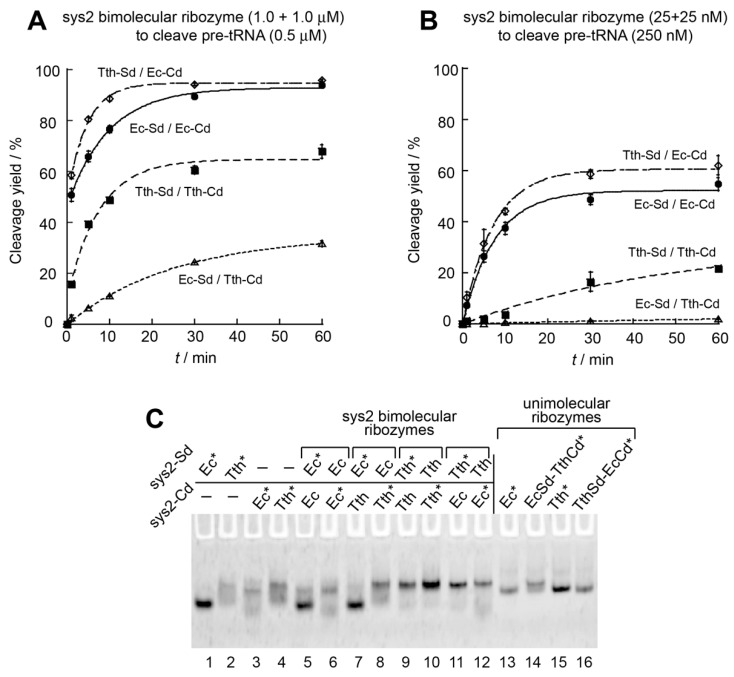
Catalytic ability and interdomain assembly of the sys2 bimolecular ribozymes. (**A**) Site-specific cleavage reactions of pre-tRNA catalyzed by twofold excess of sys2 bimolecular RNase P ribozymes. Reactions were performed in the presence of 0.5 μM pre-tRNA, 1.0 μM S-domain RNA, and 1.0 μM C-domain RNA at 37 °C. (**B**) Site-specific cleavage reactions of pre-tRNA catalyzed by a catalytic amount of sys2 bimolecular RNase P ribozymes. Reactions were performed in the presence of 250 nM pre-tRNA, 25 nM S-domain RNA, and 25 nM C-domain RNA at 37 °C. (**C**) EMSA of S-domain/C-domain complexes in the presence of 50 mM Mg^2+^. Unimolecular RNase P ribozymes were used as size markers for the complexes. Asterisks indicate RNAs labeled with the BODIPY fluorophore.

It has been reported that the catalytic ability of the *E. coli* RNase P ribozyme can be reconstituted by the bimolecular system-1 (sys1) and also system-2 (sys2) [20]. In the sys1 bimolecular ribozyme, physical affinity between the S-domain and C-domain was low and the resulting bimolecular ribozyme was hardly active (3.3% after a 60 min reaction) in the presence of twofold excess S-domain and C-domain RNAs (1.0 μM each) over substrate pre-tRNA (0.5 μM) (Figure 2) [20]. In the sys2 ribozyme, the association between the S-domain and the C-domain was improved due to P6 base pairs serving as an interdomain interaction [20]. While the sys2 ribozyme exhibited significant catalytic ability in the presence of twofold excess ribozyme (Figure 3A), the *E. coli* sys2 ribozyme did not form a stable complex in the electrophoretic mobility shift assay (EMSA) with 50 mM Mg^2+^ [20]. A possible strategy to produce improved derivatives of bimolecular bacterial RNase P ribozymes is the use of thermostable homologs of the *E. coli* ribozyme [27,30,31]. We examined an A-type RNase P ribozyme from *T. thermophilus* because the *T. thermophilus* RNase P ribozyme is a typical A-type ribozyme with a similar secondary structure to the *E. coli* ribozyme (Figure 1A,B) [27]. Furthermore, the 3D structure of the isolated S-domain of the *T. thermophilus* ribozyme has been elucidated [26]. Therefore, we constructed sys1 and sys2 bimolecular ribozymes using the *T. thermophilus* RNase P ribozyme.

We first compared the catalytic activity of the *T. thermophilus* sys1 bimolecular ribozyme with that of the *E. coli* sys1 ribozyme (Figure 2A). In addition to the *T. thermophilus* and *E. coli* sys1 ribozymes, separately prepared S-domain and C-domain RNAs enabled examination of two additional sys1 ribozymes consisting of heterologous (chimeric) pairs of S-domain and C-domain RNAs (Ec-Sd/Tth-Cd and Tth-Cd/Ec-Sd chimeric ribozymes). Among the four sys1 bimolecular ribozymes, the *E. coli* ribozyme was poorly active and the Ec-Sd/Tth-Cd chimeric ribozyme showed no activity (Figure 2A). On the other hand, the *T. thermophilus* sys1 ribozyme exhibited weak catalytic activity and the product yield reached 29% after a 60 min reaction (Figure 2A). Interestingly, the second chimeric sys1 ribozyme (Tth-Sd/Ec-Cd) was as active as the *T. thermophilus* sys1 ribozyme (Figure 2A). This observation suggested that the identity of the S-domain RNA (the *T. thermophilus* S-domain RNA) makes a positive contribution to the catalytic activity of sys1 bimolecular ribozymes. We then performed an EMSA to examine whether the S-domain RNA and the C-domain RNA, each of which showed folding uniformity to yield a single band (Figure 2B, lanes 1–4), formed a stable complex. In the presence of 50 mM Mg^2+^, the *T. thermophilus* ribozyme (lanes 9 and 10) and the Tth-Sd/Ec-Cd ribozyme (lanes 11 and 12) exhibited no stable S-domain/C-domain complexes, suggesting that the two active bimolecular sys1 ribozymes were structurally unstable (Figure 2B).

We next examined the sys2 ribozymes with four S-domain/C-domain combinations. In the presence of twofold excess ribozyme units, each sys2 ribozyme was more active than the corresponding sys1 ribozyme (Figure 3A). In the sys2 format, the *E. coli* ribozyme was more active than the *T. thermophilus* ribozyme (Figure 3A). The Tth-Sd/Ec-Cd sys2 ribozyme showed comparable activity to the *E. coli* sys2 ribozyme (Figure 3A). In the presence of twofold excess ribozyme units, an initial burst was observed in the cleavage reaction, where the *E. coli* ribozyme and the Tth-Sd/Ec-Cd chimera ribozyme cleaved 51% and 58% of the substrate pre-tRNA within 1 min, respectively (Figure 3A), suggesting that the bimolecular sys2 ribozyme complex of Tth-Sd/Ec-Cd is slightly more stable than that of the *E. coli* ribozyme. The *T. thermophilus* sys2 ribozyme showed a modest initial burst, where 16% of the substrate was cleaved (Figure 3A). To further investigate the catalytic abilities of the four sys2 ribozymes, we then examined the substrate cleavage reactions in the presence of 10-fold excess molar amount of pre-tRNA substrate (250 nM) over lower concentrations (25 nM) of S-domain and C-domain RNAs (Figure 3B). In the presence of excess substrate, the *E. coli* ribozyme and Tth-Sd/Ec-Cd ribozyme were still distinctly more active than the other two sys2 ribozymes with the *T. thermophilus* C-domain RNA (Figure 3B).

To examine whether the promising catalytic and structural properties of the Tth-Sd/Ec-Cd ribozyme were observed only in the bimolecular formats, we prepared unimolecular versions of the two chimeric ribozymes and compared their activities with the wild-type *E. coli* and *T. thermophilus* RNase P ribozymes (Figure S2). Under conditions of substrate excess, the *E. coli* ribozyme and the Tth-Sd/Ec-Cd ribozyme were distinctly more active than the other two ribozymes (Figure S2A). The Tth-Sd/Ec-Cd ribozyme was as active as the *E. coli* ribozyme (Figure 3A,B and Figure S2A). Additional comparisons among the four unimolecular ribozymes revealed that the *T. thermophilus* ribozyme preferred a reaction temperature higher than 37 °C for its full catalytic activity (Figure S2B). Unfortunately, this property was not matched with its utilization in bimolecular formats.

EMSA with 50 mM Mg^2+^ strongly suggested that the Tth-Sd/Ec-Cd sys2 ribozyme (lanes 11 and 12) and *T. thermophilus* sys2 ribozyme (lanes 9 and 10) formed complexes between their S-domain and C-domain based on mobility shift of their S-domain RNA in the presence of the C-domain RNA (Figure 3C). The other two ribozymes (*E. coli* and Ec-Sd/Tth-Cd ribozymes) showed no mobility shift in S-domain RNA (lanes 5 and 7), suggesting no stable complex formation (Figure 3A,B). It should be noted that the Tth-S domain (Figure 3C, lane 2) and the Ec-C domain (Figure 3C, lane 3) both showed broad bands in the sys2 format although the S-domain and C-domain in the sys1 format both showed sharp bands (Figure 2B, lanes 2 and 3). These observations indicated that the folding abilities of the S-domain and C-domain were disturbed by dissecting them at the junction between P5 and P6 elements. The overall folding of S-domain/C-domains, however, was recovered to give a single band as their complex when they were assembled into the bimolecular sys2 complex (Figure 3C, lanes 11 and 12). Electrophoretic mobilities of the sys2 S-domain/C-complexes of the Tth-Sd/Ec-Cd (Figure 3C, lanes 11 and 12) and *T. thermophilus* ribozymes (Figure 3C, lanes 9 and 10) were almost the same as that of the unimolecular Ec-Sd/Ts-Cd ribozyme (Figure 3C, lane 14), which was the least active among the four unimolecular ribozymes (Figure S2A). The remaining three unimolecular ribozymes (lanes 13, 15, and 16) migrated slightly faster than the unimolecular Ec-Sd/Ts-Cd ribozyme (Figure 3C). The major bands in the two sys2 bimolecular ribozymes (Figure 3C, lanes 9–12) and the Ec-Sd/Tth-Cd unimolecular ribozyme (Figure 3C, lane 13) may correspond to their folding intermediates, the structures of which are less compact than their fully folded states.

### 3.2. Modular Engineering of L9–P1 Interdomain Interactions

The Tth-Sd/Ec-Cd chimeric RNase P ribozyme consisting of the *T. thermophilus* S-domain and the *E. coli* C-domain is promising for utilization as a bimolecular ribozyme. We optimized the modular parts of the Tth-Sd/Ec-Cd ribozyme using the sys1 format, with which we can readily monitor the improvement of catalytic ability due to the modest activity of the parent sys1 ribozymes (Figure 2). We examined the optimization of L9–P1 interaction [32,33,34] utilizing a GNRA tetraloop–receptor interaction, which is a class of modular tertiary interactions between tetraloops consisting of a GNRA consensus sequence (N = any of the four bases; R = purine) and their receptor motifs and observed most frequently and widely in naturally occurring RNA structures [35,36,37]. The receptor motifs in the *E. coli* and *T. thermophilus* P1 elements are classified as primitive forms of GNRA receptors, the binding affinity of which has not been optimized over evolution [37].

**Figure 4:**
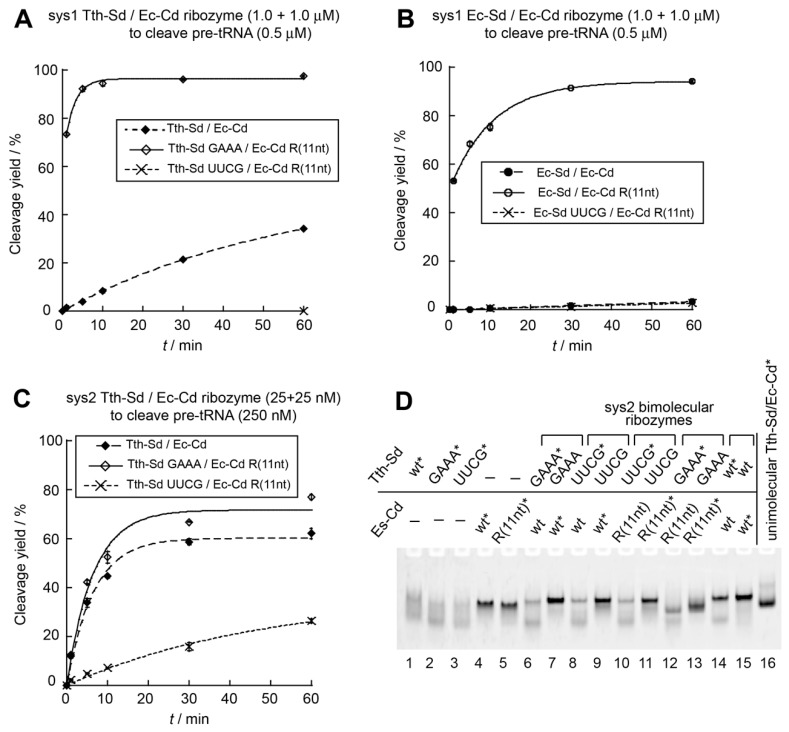
Effects of L9–P1 tertiary interaction on catalytic ability and interdomain assembly of the bimolecular ribozymes. (**A**) Site-specific cleavage reactions of pre-tRNA catalyzed by twofold excess of sys1 Tth-Sd/Ec-Cd bimolecular RNase P ribozymes. Reactions were performed in the presence of 0.5 μM pre-tRNA, 1.0 μM S-domain RNA with L9 tetraloop, and 1.0 μM C-domain RNA with P1 tetraloop receptor. (**B**) Site-specific cleavage reactions of pre-tRNA catalyzed by twofold excess of sys1 *E. coli* bimolecular ribozymes. Reactions were performed in the presence of 0.5 μM pre-tRNA, 1.0 μM S-domain RNA with L9 tetraloop, and 1.0 μM C-domain RNA with P1 tetraloop receptor. (**C**) Site-specific cleavage reactions of pre-tRNA catalyzed by catalytic amount of sys2 Tth-Sd/Ec-Cd bimolecular ribozymes. Reactions were performed in the presence of 250 nM pre-tRNA, 25 nM S-domain RNA with L9 tetraloop, and 25 nM C-domain RNA with P1 tetraloop receptor. (**D**) EMSA of S-domain/C-domain complexes in the presence of 50 mM Mg^2+^. Unimolecular RNase P ribozymes were used as size markers for the complexes. Asterisks indicate RNAs labeled with the BODIPY fluorophore.

We substituted the L9 loop and P1 receptor pairs in the four sys1 ribozymes with the most advanced form consisting of a GAAA loop and the R(11nt) receptor motif. The R(11nt) receptor motif specifically and strongly recognizes a GAAA loop [36]. In the sys1 bimolecular system, the variant Tth-Sd/Ec-Cd chimeric ribozyme possessing L9-GAAA loop and P1-R(11nt) receptor showed marked improvement of the activity (Figure 4A). Under conditions of ribozyme excess where the parent Tth-Sd/Ec-Cd sys1 ribozyme was modestly active (Figure 2A and Figure 4A), its L9-GAAA/P1-R(11nt) variant was highly active because it exhibited a large initial reaction burst, with which 73% of the substrate was cleaved within the first 1 min (Figure 4A). This was in marked contrast to the parent Tth-Sd/Ec-Cd sys1 ribozyme, which showed no initial burst phase (Figure 4A). The functional importance of the L9–P1 interaction was supported by its complete disruption by replacing the L1-GAAA loop with a UUCG loop. The L9-UUCG variant yielded no detectable amount of the product tRNA even after 60 min of incubation (Figure 4A).

Installation of the GAAA–R(11nt) pair also significantly improved the activities of the remaining three sys1 ribozymes (Figure 4B and Figure S3). In the *E. coli* sys1 bimolecular ribozyme, the variant bearing the GAAA–R(11nt) pair cleaved 58% of the substrate pre-tRNA in the initial burst phase (Figure 4B) although the parent sys1 ribozyme was hardly active (Figure 4B). The L9-GAAA and P1-R(11nt) motif pair also improved the catalytic activities of the sys2 bimolecular ribozymes. Under conditions of substrate excess, introduction of the GAAA–R(11nt) pair to the Tth-Sd/Ec-Cd sys2 ribozyme further improved its activity, whereas complete disruption of the L9–P1 interaction by L9-UUCG loop significantly reduced the sys2 ribozyme activity (Figure 4C). Modular engineering of the L9–P1 interaction improved the catalytic ability of the sys1 and sys2 formats of the Tth-Sd/Ec-Cd chimeric ribozyme. The L9–P1 interaction may also be a promising structural element for installation of orthogonality into the assembly interface between the S-domain and C-domain. Such orthogonality in the domain–domain interface is important in the construction of RNA nanostructures containing two or more distinct ribozyme units (Figure S1) [20,21].

In EMSA, complex formation of S-domain/C-domain bearing GAAA/R(11nt) pair was confirmed by the mobility shift of the labeled RNA component in the presence of the partner RNA (Figure 4D, lanes 12 and 13). The major band observed in the presence of the S- and C-RNAs migrated faster than the parent Tth-Sd/Ec-Cd complex (Figure 4D, lanes 14 and 15) and close to the unimolecular Tth-Sd/Ec-Cd ribozyme (Figure 4D, lane 16). These observations suggested that stable L9–P1 interaction by the GAAA/R(11nt) pair brought the sys2 ribozyme in the native gel to the fully folded state. The UUCG variant of the Tth-Sd RNA, which disrupted the L9–P1 interaction completely, also formed a complex with the Es-Cd RNA (lanes 8 and 9) or its R(11nt) variant (Figure 4D, lanes 10 and 11), the mobilities of which were similar to the Tth-Sd/Ec-Cd complex (Figure 4D, lanes 14 and 15). These observations suggested that the L9–P1 interaction was not stable in the parent Tth-Sd/Ec-Cd sys2 complex and it existed predominantly as a folding intermediate in the native gel.

### 3.3. Engineering of P6 Base Pair Interaction in the Sys2 Ribozyme

**Figure 5:**
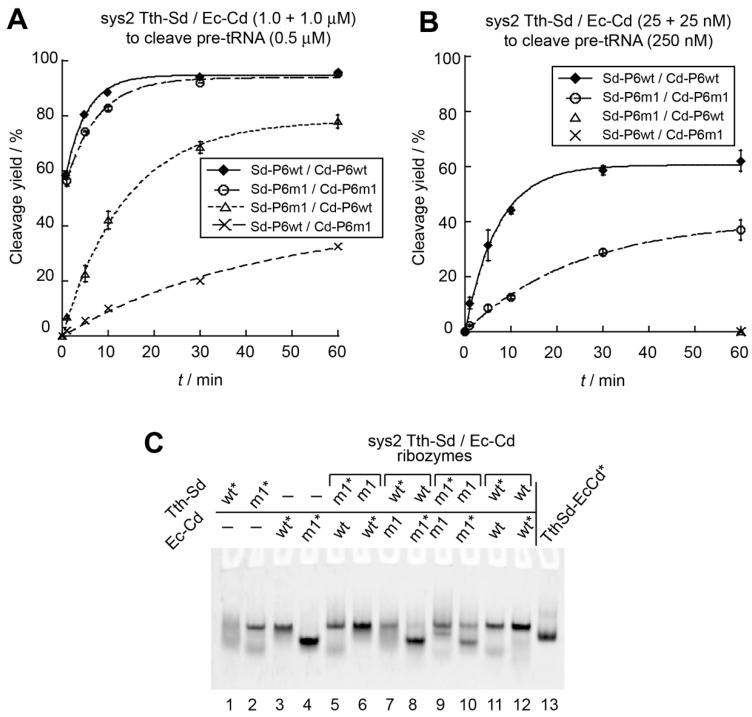
Effects of P6 base pairs on catalytic ability and interdomain assembly of the sys2 Tth-Sd/Ec Cd bimolecular ribozymes. (**A**) Site-specific cleavage reactions of pre-tRNA catalyzed by twofold excess of sys2 Tth-Sd/Ec-Cd bimolecular ribozymes. Reactions were performed in the presence of 0.5 μM pre-tRNA, 1.0 μM S-domain RNA, and 1.0 μM C-domain RNA. (**B**) Site-specific cleavage reactions of pre-tRNA catalyzed by catalytic amount of sys2 Tth-Sd/Ec-Cd bimolecular ribozymes. Reactions were performed in the presence of 250 nM pre-tRNA, 25 nM S-domain RNA, and 25 nM C-domain RNA. (**C**) EMSA of S-domain/C-domain complexes of sys2 Tth-Sd/Ec-Cd ribozymes in the presence of 50 mM Mg^2+^. Asterisks indicate RNAs labeled with the BODIPY fluorophore.

In the Tth-Sd/Ec-Cd sys2 bimolecular ribozyme, we next engineered the P6 base pairs, which provide interdomain interaction, to generate an orthogonal S-domain/C-domain interaction (Figure S1). A compensatory mutation experiment was performed using the *E. coli* sys2 ribozyme, in which the catalytic ability of the *E. coli* sys2 ribozyme was supported by mutant P6 base pairs (P6m1), whereas mismatched P6 base pairs significantly reduced the activity. Therefore, we introduced the P6m1 base pairs into the Tth-Sd/Ec-Cd sys2 ribozyme (Figure 1C). Under conditions of ribozyme excess, the activity of a variant Tth-Sd/Ec-Cd ribozyme with the P6m1 base pairs was close to that of the parent ribozyme. The initial burst (56% of substrate cleavage) of the P6m1 variant was close to that of the parent Tth-Sd/Ec-Cd sys2 ribozyme (58% of substrate cleavage) (Figure 5A). A mutant sys2 ribozyme with a mismatched P6 element with P6m1 in the S-domain and the original P6 in the C-domain was moderately active (Figure 5A). The second mutant possessing the original P6 in the S-domain and P6m1 in the C-domain retained weak catalytic activity, which was lower than that of the first mutant (Figure 5A). It should be noted that the two mismatched mutants both showed no initial reaction burst, suggesting that their bimolecular complexes may be destabilized by mismatched P6 base pairs.

In the presence of a 10-fold excess amount of the substrate pre-tRNA, the variant sys2 ribozyme with P6m1 was 3.5-fold less active than the parent Tth-Sd/Ec-Cd sys2 ribozyme (Figure 5B). Under these conditions, the two mutants with mismatched P6 base pairs yielded no detectable amount of product tRNA even after a 60 min reaction (Figure 5B). These observations indicated that the assembly of the S-domain and C-domain RNAs cannot be supported without matched P6 base pairs under low concentrations of domain RNAs (25 + 25 nM).

While P6m1 base pairs supported the catalytic activity of the Tth-Sd/Ec-Cd sys2 ribozyme, P6m1 appeared to perturb folding of S-domain and C-domain RNAs because gel mobilities of the *T. thermophilus* S-domain RNAs (Figure 5C, lanes 1 and 2) and the *E. coli* C-domain RNAs (Figure 5C, lanes 3 and 4) showed significant differences between the parent RNA and P6m1 variant. The bimolecular complex bearing P6m1 base pairs, however, produced a band (Figure 5C, lanes 9 and 10), the mobility of which was not different from that of the parent Tth-Sd/Ec-Cd complex (Figure 5C, lanes 11 and 12). These results suggested that in the sys2 format Tth-Sd/Ec-Cd chimeric ribozyme, modulation of the P6 base pairs can be utilized to produce an orthogonal interface between the S-domain and the C-domain. To generate an ideal pair of bimolecular RNase P ribozymes, assembly of S-domain/C-domain should be highly orthogonal to each other and the two ribozymes should have similar activities (Figure S1). This required additional modification of the sys2 ribozyme.

### 3.4. Construction an Orthogonal Pair of Sys2 Bimolecular Ribozymes with Improved Catalytic Properties

**Figure 6:**
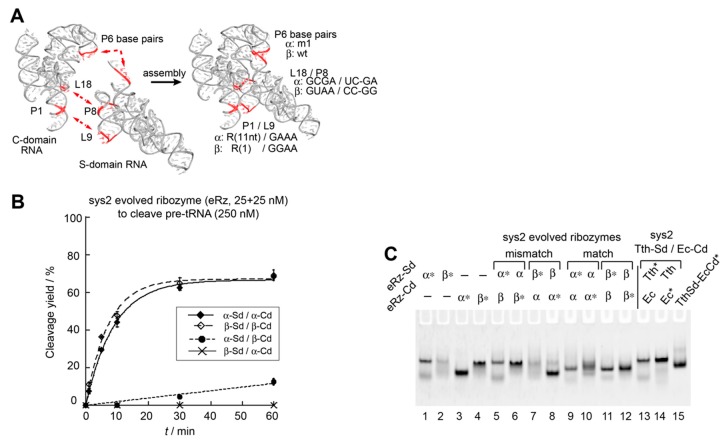
A pair of sys2 bimolecular ribozymes with orthogonal domain–domain assembly. (**A**) Three interdomain interactions assembling S-domain and C-domain in the sys2 ribozyme are shown on the left. The identities of three interactions in α and β variants are shown on the right. (**B**) Site-specific cleavage reactions of pre-tRNA catalyzed by catalytic amount of sys2 α and β variant ribozymes. Reactions were performed in the presence of 250 nM pre-tRNA, 25 nM S-domain RNA, and 25 nM C-domain RNA. (**C**) EMSA of S-domain/C-domain complexes of sys2 α and β variant ribozymes in the presence of 50 mM Mg^2+^. Asterisks indicate RNAs labeled with the BODIPY fluorophore.

In the Tth-Sd/Ec-Cd sys2 ribozyme, we demonstrated that modifications of L9–P1 interaction (Figure 4) and P6 base pairs (Figure 5) were useful to improve the catalytic activity and/or control S-domain/C-domain assembly. We used these modifications simultaneously to generate an improved pair of orthogonal S-domain/C-domain interfaces. In addition to L9–P1 interaction and P6 base pairs, we additionally engineered a third interdomain interaction (P8–L18) in the Tth-Sd/Ec-Cd sys2 ribozyme (Figure 1). The P8–L18 interaction utilizes a primitive-type GNRA loop–receptor interaction consisting of L18-GCGA loop in the C-domain and P8 UC–GA base pairs in the S-domain (Figure 1D). This interaction can be isosterically interchangeable with another primitive GUAA/CC–GG pair (Figure 1D) [35,36,37]. Although the selectivity and affinity of the two primitive pairs may not be strong, they are expected to assist in differentiation of the pair of S-domain/C-domain interfaces (Figure 6A). In addition to introduction of the GAAA/R(11nt) pair in the L9–P1 interaction, we used the GGAA/R(1) pair to substitute the original L9–P1 with the primitive GNRA/receptor pair. The GGAA/R(1) pair belongs to the evolved form of GNRA/receptor pairs, which have high affinity and specificity in their recognition due to internal loops in the receptors [37,38]. The recognition properties of the GGAA/R(1) pair were similar to those of the GAAA/R(11nt) pair, while the two evolved loop/receptor pairs were orthogonal to each other [38]. We designed a variant termed ebRz-α (evolved bimolecular ribozyme-α) (Figure 1C,D and Figure 6A) based on the Tth-Sd/Ec-Cd sys2 ribozyme. The ebRz-α ribozyme possessed P6m1 base pairs, GAAA/R(11nt) pair in L9–P1, and GCGA/UC-GA pair in P8–L18. We also designed a second variant, which served as an orthogonal partner of ebRz-α. The second variant, designated as ebRz-β (evolved bimolecular ribozyme-β), possessed the wild-type P6 pair, GGAA/R(1) pair in L9–P1, and GUAA/CC-GG pair in P8–L18 (Figure 1C,D and Figure 6A). Between the ebRz-α and ebRz-β ribozymes, all three interdomain interactions (P8–L18, P1–L9, and P6 base pairs) were differentiated from each other.

We examined the catalytic abilities of the ebRz-α and ebRz-β ribozymes and also the orthogonality between their S-domain/C-domain interfaces. Under conditions of substrate excess, ebRz-α and ebRz-β exhibited similar activities (Figure 6B and Figure S4). Their activities were similar to the variant Tth-Sd/Ec-Cd sys2 ribozyme with GAAA/R(11nt) interaction in L9–P1 (Figure 5B). The substrate pre-tRNA was cleaved inefficiently in the presence of mismatched S-domain/C-domain combinations of ebRz-α and ebRz-β (Figure 6B). A mismatched combination with the ebRz-α S-domain and ebRz-β C-domain showed *k*_obs_ = 0.002 min^−1^, which was 37-fold and 49-fold lower than those of matched ebRz-α (0.074 min^−1^) and ebRz-β (0.098 min^−1^), respectively (Figure 6B). The second mismatched combination with the ebRz-β S-domain and ebRz-α C-domain yielded no detectable amount of the product (Figure 6B). These results indicated that the S-domain/C-domain assemblies of the ebRz-α and ebRz-β ribozymes were considerably orthogonal to each other. Complex formation between S-domain and C-domain was observed in the ebRz-α and ebRz-β ribozymes (Figure 6C, lanes 9–12). In the two mismatched combinations (Figure 6C, lanes 5–8), no changes were observed in the mobility of a fluorophore-labeled RNA component between the presence and absence of its mismatched partner component, suggesting that no stable complex was formed between the mismatched S-domain RNA and C-domain RNA of the evolved ribozymes.

## 4. Discussion

This study was performed to investigate A-type bacterial RNase P ribozyme from a thermophilic bacterium (*T. thermophilus* ribozyme) to determine whether its bimolecular format is promising as a platform for the development of supramolecular ribozyme polymers. Comparative analysis of the bimolecular *T. thermophilus* and *E. coli* ribozymes indicated that a chimeric bimolecular ribozyme consisting of the *T. thermophilus* S-domain RNA and *E. coli* C-domain (Tth-Sd/Ec-Cd sys2 ribozyme) had promising properties related to catalytic ability and complex formation. In EMSA analysis, no S-domain/C-domain complex of the *E. coli* sys2 ribozyme was observed (Figure 3C, lanes 5 and 6) although its catalytic activity was comparable to that of Tth-Sd/Ec-Cd sys2 ribozyme (Figure 3A–C and Figure S2A). This observation suggests that the active complex of the *E. coli* sys2 ribozyme may be induced by the association with pre-tRNA. The complex was detected in the *T. thermophilus* sys2 ribozyme (Figure 3C, lanes 9 and 10) although the catalytic activity of the *T. thermophilus* sys2 ribozyme was less efficient than that of the *E. coli* sys2 especially under conditions of substrate excess (Figure 3B). These results were reasonable because RNase P ribozymes from several thermophilic bacteria were more active than the *E. coli* ribozyme at 50–60 °C but they were often less active than the *E. coli* ribozyme at 30–40 °C. The relatively poor activities of thermophilic RNase P ribozymes at 30–40 °C are partially due to slow product release [30]. The interaction between the product RNA and RNase P ribozymes at 37 °C may also conduct substrate inhibition, with which reactions reached plateau before complete consumption of the pre-tRNA substrate. These results suggest that their RNA structures are too rigid at 30–40 °C because they are adapted to the high-temperature conditions of thermophilic bacterial habitats.

The *T. thermophilus* ribozyme formed a stable S-domain/C-domain complex, while the *E. coli* ribozyme was distinctly more active at 37 °C. This was largely resolved in the chimeric Tth-Sd/Ec-Cd sys2 ribozyme because it formed a stable complex and exhibited promising catalytic activity. Although we did not exploit the molecular mechanism of action of the Tth-Sd/Ec-Cd sys2 ribozyme to exhibit promising structural and catalytic properties, these properties may be due to the *T. thermophilus* S-domain, which is rigid at 37 °C, and the *E. coli* C-domain, which must adapt its catalytic ability at 37 °C. Further improvement of the bimolecular Tth-Sd/Ec-Cd sys2 ribozyme was achieved by optimizing the interdomain tetraloop/receptor interaction between L9 in the S-domain and P1 in the C-domain. Modulation of tetraloop/receptor interactions has been employed successfully to redesign the structural and catalytic properties of group I intron ribozymes [21]. This strategy, however, has been poorly applied to biochemical studies of RNase P ribozymes [32]. This study demonstrated that tetraloop/receptor interactions can also represent modular parts useful for engineering and redesign of RNase P ribozymes.

Installation of orthogonality to the RNA–RNA interface between the S-domain and C-domain relied mainly on the substitution of base pairs in the P6 element and was also assisted by the two sets of tetraloop/receptor interactions (Figure 6). The resulting pair of sys2 bimolecular ribozymes (ebRz-α and ebRz-β) are promising components for the development of supramolecular ribozyme copolymers derived from RNase P ribozymes (Figure S1). This study will contribute to the construction of RNase P ribozyme-based homopolymers and copolymers (Figure S1), although a number of construction issues remain to be resolved. One such issue is RNA folding of the S-domain and C-domain of the sys2 bimolecular ribozymes. The migration patterns of the S-domain and C-domain in EMSA were strongly influenced by structural engineering, such as conversion of the sys1 format to the sys2 format (Figure 2B and Figure 3C) and substitutions at the P6 element to produce P6m1 (Figure 5C). In the sys2 format, the secondary and tertiary structures of the isolated S-domain and C-domain RNAs may not be robust against these structural modifications. Folding of the S-domain and C-domain may become more serious problems when they are artificially reconnected to produce a unit RNA for ribozyme oligomers based on the sys2 Tth-Sd/Ec-Cd ribozyme. We will continue our efforts to improve the folding properties of the Tth-Sd/Ec-Cd sys2 ribozyme and its derivatives developed in this study. We are also searching for RNase P ribozymes or their S- and/or C-domains that are robust to structural engineering for use in the construction of RNase P ribozyme-based nanostructures. Such RNase P ribozyme-based nanostructures may be applicable to RNase P-based gene targeting therapy if they can be constructed in living cells [39,40]. Because their catalytic activity can be regulated more stringently than that of monomeric ribozymes depending on the concentration of the ribozyme RNA, RNase P ribozyme-based nanostructures may serve as an improved form of RNA-based nanobiotechnology tools.

## 5. Conclusions

A heterologous S-domain/C-domain combination consisting of *T. thermophilus* S-domain RNA and *E. coli* C-domain showed better properties with regard to assembly and catalysis than homologous S-domain/C-domain combinations. The assembly and catalytic properties of the chimeric ribozyme were improved by installation of the evolved form of GNRA/receptor interaction. Based on these modifications, a pair of orthogonal bimolecular RNase P ribozyme derivatives was developed. Although problems remain to be resolved, these derivatives would serve as advanced forms of RNase P ribozyme for the construction of catalytic RNA nanostructures. The strategies described in this study will be applicable to the engineering of other functional RNAs to elucidate their structure–function relationships and also for their application as RNA nanobiotechnology tools.

## Supplementary Materials

The following are available online at https://www.mdpi.com/2079-7737/8/3/65/s1, Figure S1: Schematic outline of the design of one-dimensional (1D) ribozyme assemblies, in which bimolecular ribozymes serve as functional units; Figure S2: Catalytic ability of the unimolecular RNase P ribozymes; Figure S3: Effects of L9–P1 tertiary interaction on catalytic ability and interdomain assembly of the bimolecular ribozymes; Figure S4: Effects of Mg^2+^ concentration on the evolved sys2 bimolecular ribozymes.
