# Supplementary material for: Rational Design of an Orthogonal Pair of Bimolecular RNase P Ribozymes through Heterologous Assembly of Their Modular Domains
Source: Biology (Basel). 2019 Aug 31;8(3):65. doi: 10.3390/biology8030065 (PMC6783828; doi:10.3390/biology8030065)
Supplement: Supplementary file 1 [file biology-08-00065-s001.zip › rev-Nozawa_2nd_SI.pdf]

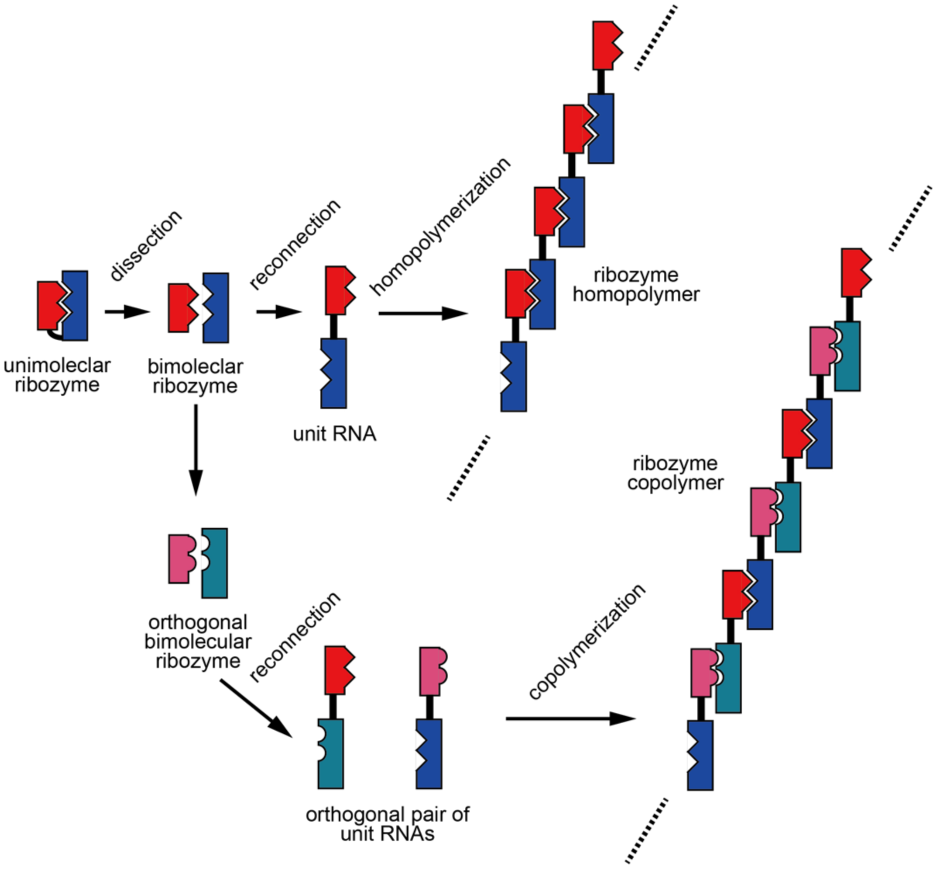

Figure S1

Schematic outline of the design of one-dimensional (1D) ribozyme assemblies, in which bimolecular ribozymes serve as functional units. A 1D ribozyme homopolymer was formed by one unit RNA, whereas a 1D ribozyme homopolymer was formed by a pair of distinct bimolecular ribozyme units with orthogonal domain–domain interfaces.

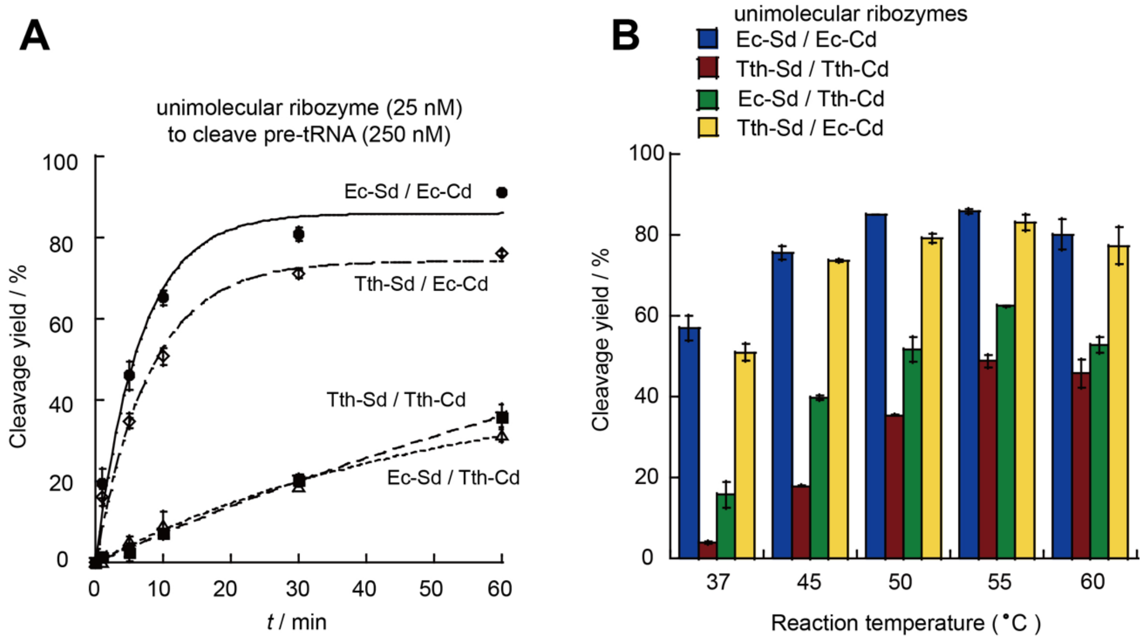

Figure S2

Catalytic ability of the unimolecular RNase P ribozymes.

A) Site-specific cleavage reactions of pre-tRNA catalyzed by a catalytic amount of the unimolecular RNase P ribozymes. Reactions were performed in the presence of 250 nM pre-tRNA and 25 nM RNase P RNA at 37°C.

B) Temperature dependence of the catalytic abilities of unimolecular RNase P ribozymes. An aqueous solution containing the unimolecular ribozyme (final concentration: 25 nM) and pre-tRNA (final concentration: 250 nM) was heated at 85°C for 5 minutes and then allowed to cool to ambient temperature. Each reaction was initiated by adding the 3× concentrated reaction buffer (final concentrations: 50 mM Tris-HCl, pH 7.5, 1 M KCl, and 50 mM $MgCl_2$) to the solution and the resulting mixtures were incubated for 20 minutes at 37°C.

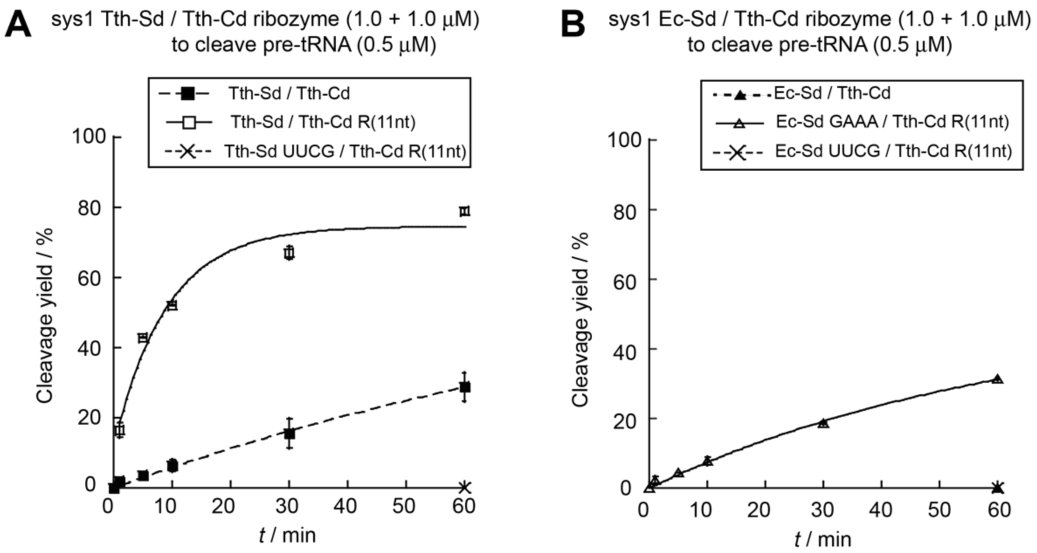

Figure S3

Effects of L9–P1 tertiary interaction on catalytic ability and interdomain assembly of the bimolecular ribozymes.

A) Site-specific cleavage reactions of pre-tRNA catalyzed by twofold excess of sys1 *T. th* bimolecular RNase P ribozymes. Reactions were performed in the presence of 0.5 μM pre-tRNA, 1.0 μM S-domain RNA with L9 tetraloop, and 1.0 μM C-domain RNA with P1 tetraloop receptor.

B) Site-specific cleavage reactions of pre-tRNA catalyzed by twofold excess of sys1 Ec-Sd/Tth-Cd bimolecular ribozymes. Reactions were performed in the presence of 0.5 μM pre-tRNA, 1.0 μM S-domain RNA with L9 tetraloop, and 1.0 μM C-domain RNA with P1 tetraloop-receptor.

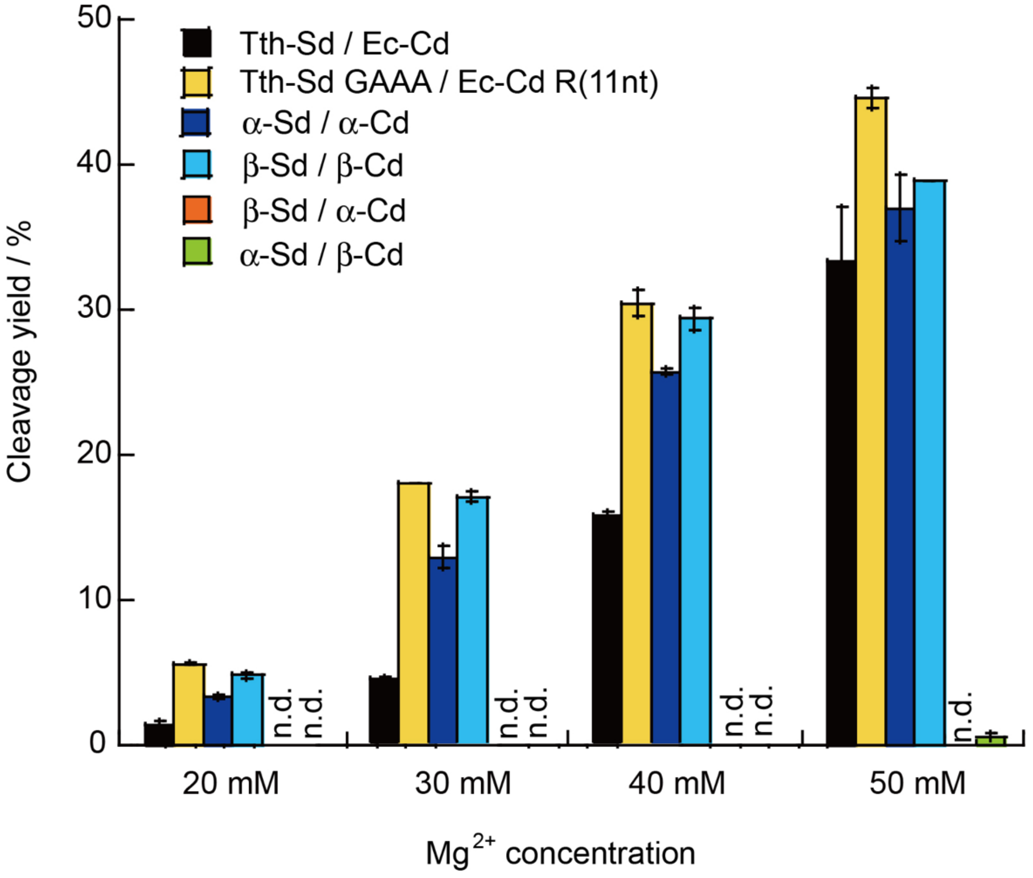

Figure S4

Effects of Mg^{2+} concentration on the evolved sys2 bimolecular ribozymes. An aqueous solution containing the unimolecular ribozyme and pre-tRNA was heated at 85°C for 5 minutes and then allowed to cool to ambient temperature. Each reaction was initiated by adding the 3× concentrated reaction buffer (final concentrations: 50 mM Tris-HCl, pH 7.5, 1 M KCl, and given concentration of $MgCl_2$) to the solution and the resulting mixture was incubated for 20 minutes at 37°C.
